# Usefulness of autofluorescence bronchoscopy in early diagnosis of airway complications after lung transplantation

**DOI:** 10.1038/s41598-020-79442-4

**Published:** 2020-12-18

**Authors:** Paolo Mendogni, Rosaria Carrinola, Lorenzo Gherzi, Davide Tosi, Alessandro Palleschi, Ilaria Righi, Francesco Damarco, Letizia Corinna Morlacchi, Gianluca Bonitta, Valentina Vaira, Mario Nosotti, Lorenzo Rosso

**Affiliations:** 1 Thoracic Surgery and Lung Transplant Unit, Fondazione IRCCS Ca’ Granda Ospedale Maggiore Policlinico, University of Milan, Via Francesco Sforza, 35, 20122 Milan, Italy; 2 Department of Pathophysiology and Transplantation, University of Milan, Milan, Italy; 3 Respiratory Unit and Adult Cystic Fibrosis Center, Department of Internal Medicine, Fondazione IRCCS Ca’ Granda Ospedale Maggiore Policlinico, Milan, Italy; 4 Division of Pathology, Fondazione IRCCS Ca’ Granda Ospedale Maggiore Policlinico, Milan, Italy

**Keywords:** Outcomes research, Respiration

## Abstract

Despite the promising results achieved so far in long-term survival after lung transplantation (LuTx), airway complications (ACs) still arise in the post-operative period. Early diagnosis and prompt treatment of ACs play a critical role in preventing their onset. Specifically, large bronchi ischemia has been recognized as a triggering factor for ACs. Autofluorescence bronchoscopy, which was first introduced for early cancer diagnosis, displays ischemic mucosae as red spots, while normal vascularized mucosae appear in green. The aim of this study is to investigate whether a significant correlation exists between ACs and the red/green (RG) ratio detected on scheduled autofluorescence bronchoscopy up to 1 year after LuTx. This prospective, observational, single-center cohort study initially considered patients who underwent LuTx between July 2014 and February 2016. All patients underwent concomitant white-light and autofluorescence bronchoscopy at baseline (immediately after LuTx), on POD7, POD14, POD21, POD28, POD45, 3 months, 6 months, and 1 year after LuTx. An autofluorescence image of the first bronchial carina distal to the anastomosis was captured and analyzed using histograms for red and green pixels; the R/G ratio was then recorded. Potential ACs were classified according according to the presence of a white-light following the MDS (macroscopic aspect, diameter and suture) criteria. The authors assessed the association between the R/G ratio and the ACs occurrence using a generalized estimating equations model. Thirty-one patients met the inclusion criteria and were therefore selected. Out of a total of 53 bronchial anastomoses, 8 developed complications (late bronchial stenosis), with an average onset time of 201 days after LuTx. ACs showed a similar baseline covariate value when compared to anastomoses that involved no complication. Generalized estimating equations regression indicated a clear association over time between the R/G ratio and the rise of complications (*p* = 0.023). The authors observed a significant correlation between post-anastomotic stenosis and the delayed decrease of the R/G ratio. Preliminary outcomes suggest that autofluorescence bronchoscopy may be an effective and manageable diagnostic tool, proving complementary to other instruments for early diagnosis of ACs after LuTx. Further research is needed to confirm and detail preliminary findings.

## Introduction

Nowadays, LuTx remains the final therapeutic option for end-stage pulmonary disease in selected patients. Nevertheless, its establishment as standard practice is relatively recent, making that of LuTx a field still awaiting exploration, if compared to other solid organ transplant procedures.

Up to the present day, remarkable results have been achieved in terms of long-term survival and quality of life, improving immunosuppression, and perioperative management. Even so, patients’ lives are faced with an increased risk of complications during both early and late post-transplant period.

ACs usually result in multiple (adverse) events in the shorter term, while chronic rejection and bronchiolitis obliterans syndrome usually develop in the longer term^1^. ACs do not only depend on the surgical anastomotic technique, as the healing of the suture is impacted by several variables related to the donor, the recipient and the graft, primarily age, gender, infections, mechanical ventilation and cardiocirculatory support, graft ischemic time, and intensive care unit (ICU) stay^2^.

The incidence of bronchial complications widely varies and ranges between 7 and 18% with a mortality rate between 2 and 5%^3^. In addition, post-transplant ACs may occur in the late or post-operative period; ACs may involve the anastomosis or the distal airways, and include varying degrees of ischemia and necrosis, or evolve to dehiscence, stricture (stenosis) or bronchomalacia.

In spite of the current body of knowledge, the early diagnosis and classification of ACs are not always straightforward. It is a fact that early detection of ACs and consequent therapeutic interventions drastically affect post-operative outcomes and patients’ life expectancy after LuTx.

Autofluorescence Imaging (AFI) can be performed in conjunction with conventional white-light endoscopy, as it has been demonstrated that it can enhance overall specificity and sensitivity up to 85% and 90% respectively, in recognizing early mucosal alterations^4,5^.

The aim of the present study is to determine whether a correlation exists between ACs and the R/G ratio detected by autofluorescence bronchoscopy over time in early LuTx follow up.

## Materials and methods

This prospective, observational, single-center cohort study considered patients submitted to lung transplantation at Foundation IRCCS Ca’ Granda Ospedale Maggiore Policlinico of Milan, between July 2014 and February 2016.

The inclusion criteria were: age range between 18 and 65 years; single or bilateral sequential lung transplantation; availability of specific flexible bronchoscope with autofluorescence immediately after transplantation and during the study time-points; informed consent signed by patients. Exclusion criteria were: grafts derived from donors after cardiac death (DCD); lung re-transplantation and ICU stay > 7 days. Ethical Committee approval: Fondazione IRCCS Ca’ Granda Ospedale Maggiore Policlinico, Milan, Italy (Ref n. 181, 24 January 2017). The Authors declare that no organs were procured from prisoners. Furthermore, all organs were procured whilst respecting the patients’ privacy. All procedures were carried out in accordance with relevant guidelines and regulations.

### Organ retrieval and preservation

All grafts were retrieved from brain-death donors (DBD). Lung procurements were performed following standard procedure: after pulmonary artery cannulation, anterograde flush, aortic clamping, cardiectomy and *en bloc* double lung retrieval, lungs were divided on the back table. Grafts received anterograde and retrograde flushes with a low-potassium dextran glucose solution (Perfadex). During transport, grafts were stored on ice. When necessary, grafts underwent ex-vivo lung perfusion (EVLP) following our previously reported protocol^6^, and received another flush with the low-potassium dextran glucose solution at the end of the procedure. Afterwards, lungs were stored on ice until transplantation.

### Lung transplantation

Lung transplantations were performed with a standardized technique that saw no variation during the study period. In particular, bilateral lung transplantations were sequential, with two main bronchi anastomoses. The surgical staff did not change during the study period. Four surgeons performed all lung transplantations during the study period. Most surgical incisions were bilateral anterior thoracotomies. A Clamshell incision was only performed if specific technical difficulties arose during surgery or if central cannulation was required for extracorporeal membrane oxygenation (ECMO). The main bronchi were cut as proximally as possible to the first bronchial carina; then, when possible, bronchial “end-to-end” anastomoses were performed. Alternatively, a telescopic suture was performed in selected cases, specifically when bronchial lumen mismatch did not enable direct apposition of the graft and the bronchial stumps. The bronchial suture was performed with two running absorbable monofilament sutures (4/0 Poly-p-dioxanone) and covered with native peribronchial tissue as extensively as possible, in order to prevent ischemic damage. In case of size mismatch between donor and recipient or lung injury (i.e. lung contusions, vascular injuries), graft downsizing is usually entails atypical lung resection or, if necessary, graft segmentectomy or lobectomy, as previously described^7–9^. After LuTx, all patients were treated with the same immune suppression therapy. No induction therapy was administered in the study period. The antibiotic therapy was standardized if prophylactic, whereas it was culture-driven in case of documented bronchial colonization^10^.

### Autofluorescence imaging and white-light bronchoscopy protocols

All patients underwent concomitant white-light and AFI bronchoscopy at baseline (immediately after LuTx in the operating theatre after shifting from tracheal double-lumen tube to single-lumen tube), post-operative day POD7, POD14, POD21, POD28, POD45, 3 months, 6 months, and 1 year after LuTx. Anastomoses were classified according to the MDS grading system; for the purpose of the current study, we considered complicated anastomoses as rated higher than M0D0S0^11^. The EVIS LUCERA SPECTRUM endoscopic video imaging system combined with BF-F260 autofluorescence video bronchoscope (Olympus Medical Systems Corp.) was employed to evaluate the conditions of patients’ post-transplant airways^12^. AFI shows normal vascularized bronchial mucosa as green colored, while the ischemic mucosa as an intense red color^13^. We obtained an autofluorescence image of the first bronchial carina distally to each anastomosis at sequential time-points. The images were analyzed using histograms for red and green pixels, obtained with a commercial raster graphics editor (Adobe inc., California, U.S.); the R/G ratio was recorded.

**Figure 1:**
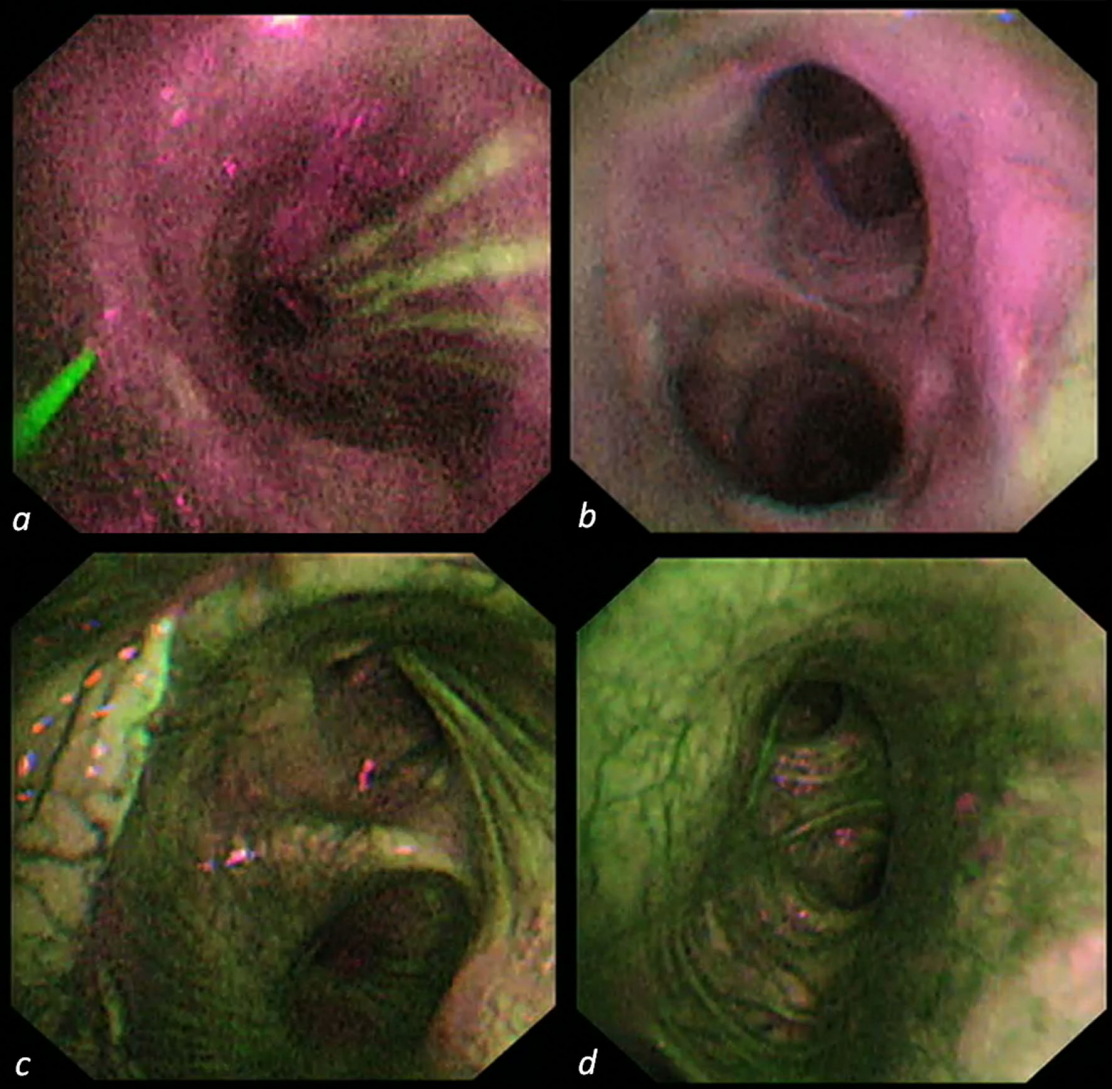
AFI appearance of first bronchial carina after bronchial anastomosis at baseline (**a**), POD28 (**b**), 6 months (**c**) and 1 year (**d**) after LuTx.

The same physician (RC) performed all procedures. Figure 1 shows AFI bronchoscopy at different time-points after LuTx.

### Statistical analysis

We considered each anastomosis as a single statistical unit and stratified them according to the presence or absence of bronchial complications. Continuous data are presented as mean and standard deviation. Binary variables are shown as absolute and percentages frequencies. Absolute standardized mean difference (ASMD) was used to evaluate baseline covariates differences across study groups. A value equal to 0.25 represented cutoffs for acceptable ASMD^14^. The R/G ratio was measured at baseline and on POD7, POD14, POD21, POD28, POD45, 3 months, 6 months, and 1 year after LuTx. The repeated measure R/G ratio data were analyzed using the ‘mean response profile’ method^15^ through generalized estimating equations (GEE) by employing time as a categorical variable and logit link function. GEE standard errors were calculated with a sandwich estimator. We used the unstructured working correlation matrix selected by correlation information criterion^16^. The GEE regression model was adjusted by R/G baseline, diabetes, coronary artery disease, recipient age, donor age, recipient gender and donor gender. We opted for the GEE to obtain a population-averaged interpretation of the regression coefficients. The null hypothesis is that the difference of R/G ratio between the two study groups was constant over time. This was verified using the multivariate Wald test, testing time (POD) × group interaction in the GEE regression model. Profile likelihood Confidence intervals (CIs) at 95% confidence level were computed. Univariate Wald test for each GEE-estimated parameter was performed. Confidence intervals (CIs) were at 95% and 2-sided *p* values were calculated. A *p *value of < 0.05 was considered statistically significant. All analyses and graphs were carried out using an R software (version 3.2.2)^17^.

## Results

We performed 39 lung transplant procedures during the enrolment period. Thirty patients met the inclusion criteria and were included in the study. Twenty-three of them underwent bilateral sequential LuTx and 7 single LuTx (2 right LuTx; 5 left LuTx). Therefore, 53 bronchial anastomoses were available for examination and data analysis. Post-anastomotic bronchial stenosis developed in eight cases, with an average onset time of 201 days after LuTx. Five bronchial stenoses (62.5%) required endoscopic treatments, absorbable endobronchial prosthesis placement in 4 cases, and endobronchial pneumatic dilating in 1 case. Anastomoses were divided in two groups: an ACs group (n = 8) and a No-ACs group (n = 45), based on the onset of at least one complication.

Clinical and demographic characteristics of patients related to each anastomosis are listed in Table 1. When considering absolute standardized mean difference, significant differences between the two anastomotic groups were identified. Specifically, the ASMD exceed 0.25 in the following variables: recipient age, recipient gender, Oto score^18^, ex-vivo lung perfusion (EVLP), and extracorporeal membrane oxygenation (ECMO) post-LuTx. No significant differences were noticed in the remaining variables.

**Table 1:** Demographic and clinical characteristics of patients, stratified by groups (ACs = Airways complications; No-ACs = No airways complications), expressed as absolute standardize mean difference.

	ACs (n = 8)	No-ACs (n = 45)	ASMD
Underlying disease, CF	0.50 (0.50)	0.64 (0.48)	0.28
Diabetes	0.50 (0.54)	0.45 (0.50)	0.11
CAD	0.13 (0.35)	0.09 (0.28)	0.14
Recipient age, years	47.36 (15.59)	41.45 (13.73)	0.42
Donor age, years	44.50 (16.59)	42.96 (15.44)	0.10
Recipient gender, male	0.63 (0.48)	0.40 (0.49)	0.46
Donor gender, male	0.625 (0.48)	0.49 (0.50)	0.28
Size mismatch, ratio	1.04 (0.02)	1.05 (0.04)	0.24
Smoking	0.25 (0.46)	0.30 (0.69)	0.07
CRP, t0	0.63 (0.52)	0.60 (0.50)	0.06
BA positive, t0	0.62 (0.52)	0.66 (0.48)	0.07
Oto score	4.88 (2.64)	3.57 (2.45)	0.52
EVLP	0.38 (0.52)	0.04 (0.20)	1.45
ECMO pre	0.25 (0.43)	0.13 (0.33)	0.28
ECMO intra	0.25 (0.43)	0.34 (0.47)	0.21
ECMO post	0.38 (0.48)	0.15 (0.36)	0.47
Bronchial anastomosis, right	0.47 (0.50)	0.53 (0.50)	0.09

*ACs* airway complications, *CAD* coronary artery disease, *BA* bronchial aspirate, *CF* cystic fibrosis, *CRP* C reactive protein, *ECMO* extracorporeal membrane oxygenation, *EVLP* ex-vivo lung perfusion.

**Figure 2:**
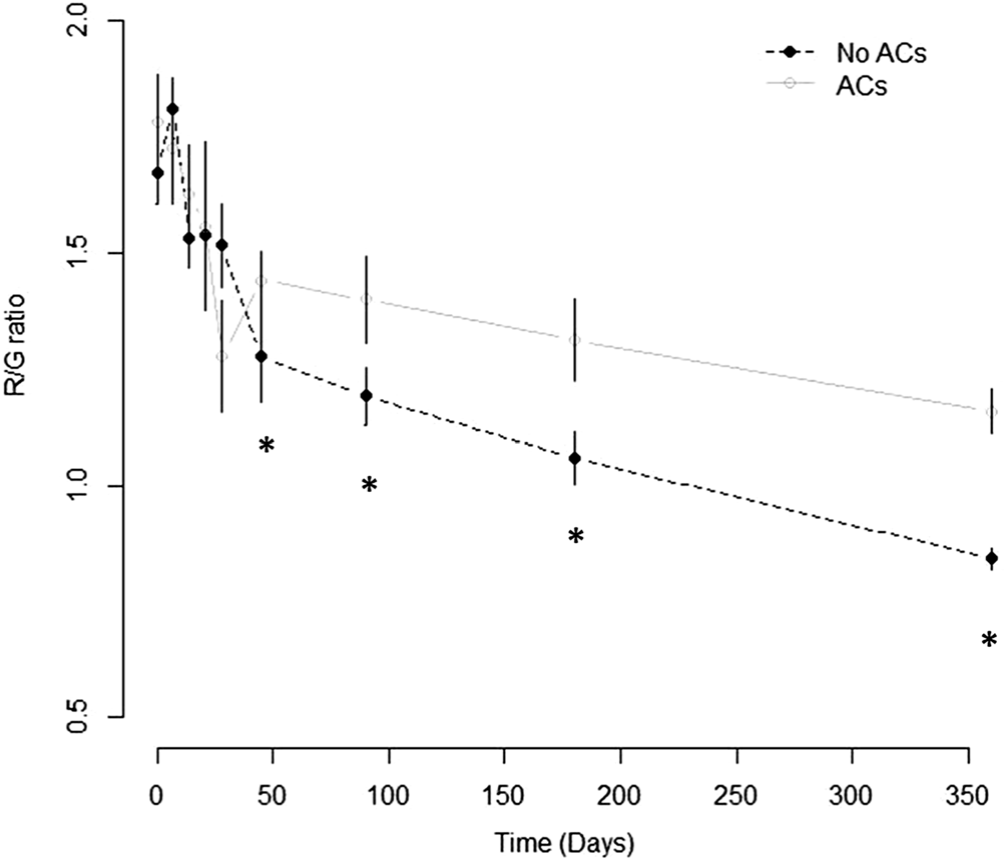
Mean R/G ratio and relative 95% error bands at baseline, POD7, POD14, POD21, POD28, POD45, 3 months, 6 months and 1 year after LuTx for the two study groups. Asterisks (*) indicated time-points with significant differences between two groups.

The mean response profiles of the R/G ratio over time for the two groups are displayed in Fig. 2.

In the GEE regression adjusted model, the omnibus test indicated that ACs and No-ACs groups were similar with respect to the baseline value (*p* = 0.147), while the “group per POD-interaction” was statistically significant (*p* = 0.023), indicating an association over time between the R/G ratio and complications (Table 2). When compared to the No-ACs group, the ACs-group displayed a significant additional 0.32 (18%) unit (with SE = 0.14, *p* = 0.024) increase in R/G ratio from baseline to POD45. In all time points before POD45, the differences in R/G ratio between the two groups were comparable (POD7, *p* = 0.221; POD14, *p* = 0.223; POD21, *p* = 0.441; POD28, *p* = 0.980). Moreover, in terms of magnitude, the R/G ratio increase was always less than half when compared to what occurred to the POD45 increment (Table 3). After POD45 the R/G ratio maintained its significance with similar magnitude over time. Furthermore, there was a time effect in the R/G ratio that decreased over time in both groups (*p* < 0.001).

**Table 2:** Wald tests of fixed effects based on an adjusted analysis of response profiles of the R/G ratio level.

Variable	DF	Chi-squared	*p* value
R/G baseline	1	3.3	0.069
POD	7	261.2	< 0.001
Group	1	2.1	0.147
Group × POD	7	16.2	0.023
Diabetes	1	10.5	0.001
CAD	1	15.2	< 0.001
Age (recipient)	1	29.4	< 0.001
Age (donor)	1	6.7	0.009
Gender (recipient)	1	18.5	< 0.001
Gender (donor)	1	2.0	0.158
CRP t0	1	11.9	< 0.001
OTO	1	8.2	0.004
EVLP	1	0.0	0.956
ECMO pre	1	5.2	0.023
ECMO intra	1	29.4	< 0.001
ECMO post	1	0.7	0.023

*CAD *coronary artery disease, *CRP* C reactive protein, *DF *degrees of freedom, *ECMO* extracorporeal membrane oxygenation, *EVLP* ex-vivo lung perfusion, *OTO* Oto score, *POD* post-operative day, *R/G* red/green ratio.

**Table 3:** Estimated GEE regression coefficients and relative 95% confidence interval based on analysis of response profiles of the R/G ratio level data at baseline, POD7, POD14, POD21, POD28, POD45, 3 months, 6 months and 1 year after LuTx.

Variable	Estimate	Standard error	95%CI	*p* value
Intercept	1.74	0.19	1.36 2.11	< 0.001
R/G baseline	1.17	0.07	0.04 0.31	0.114
POD7	0.10	0.08	− 0.03 0.16	0.168
POD 14	− 0.25	0.07	− 0.38 − 0.11	< 0.001
POD 21	− 0.29	0.07	− 0.43 − 0.15	< 0.001
POD 28	− 0.40	0.08	− 0.55 − 0.26	< 0.001
POD 45	− 0.56	0.07	− 0.69 − 0.43	< 0.001
POD 90	− 0.59	0.06	− 0.71 − 0.47	< 0.001
POD 160	− 0.72	0.06	− 0.84 − 0.60	< 0.001
POD 360	− 0.91	0.06	− 1.04 − 0.79	< 0.001
Group	0.1	0.11	− 0.03 0.25	0.334
Group × POD 7	0.16	0.13	− 0.05 0.26	0.221
Group × POD 14	0.14	0.11	− 0.09 0.36	0.223
Group × POD 21	0.12	0.15	− 0.18 0.41	0.441
Group × POD 28	0.00	0.13	− 0.25 0.26	0.980
Group × POD 45	0.32	0.14	0.04 0.60	0.024
Group × POD 90	0.26	0.13	0.01 0.51	0.039
Group × POD 160	0.31	0.14	0.04 0.58	0.027
Group × POD 360	0.35	0.13	0.09 0.60	0.008
Diabetes	− 0.27	0.05	− 0.38 − 0.16	< 0.001
CAD	− 0.42	0.06	− 0.54 − 0.31	< 0.001
Age (recipient)	0.00	0.01	− 0.01 0.01	0.427
Age (donor)	− 0.01	0.01	− 0.02 − 0.01	< 0.001
Gender (recipient)	0.26	0.08	0.09 0.42	0.002
Gender (donor)	− 0.23	0.06	− 0.35 − 0.12	< 0.001
CRP t0	0.28	0.06	0.16 0.41	< 0.001
OTO	0.08	0.02	0.05 0.12	< 0.001
EVLP	0.02	0.10	− 0.18 0.21	0.876
ECMO pre	0.49	0.21	0.08 0.88	0.018
ECMO intra	− 0.80	0.17	− 11.14 − 0.47	< 0.001
ECMO post	0.08	0.09	− 0.10 0.27	0.379
Scale parameter	0.039	0.001		

*CAD* coronary artery disease, *CI* confidence interval, *CRP *C reactive protein, *ECMO* extracorporeal membrane oxygenation, *EVLP* ex-vivo lung perfusion, *GEE* generalized estimating equations, *LuTx* lung transplantation, *OTO* Oto score, *POD* post-operative day, *R/G* red/green ratio.

## Discussion

ACs are not rare after lung transplantation^19^; their incidence has significantly decreased over time, yet they remain a major issue and a significant cause of morbidity and mortality.

The pathophysiological mechanisms of ACs after lung transplantation have been mainly linked to donor bronchial ischemia^20^, which is considered the most significant risk factor, as it affects the healing process of the bronchial anastomosis. After LuTx, the transection of the bronchial arteries at the time of lung procurement weakens the bronchi on the level of the anastomosis and the distal airway. Even though several studies have investigated the impact of bronchial arteries revascularization and omental pedicle flap, the majority of lung transplantation centers prefer more simplified techniques, e.g. surrounding the anastomosis with peribronchial tissue or intercostal muscle^21^.

The revascularization of the donor’s airways by the recipient bronchial circulation typically occurs over 2–4 weeks^22^. Thus, the viability of the donor’s bronchus heavily depends on the retrograde, low-pressure blood flow from the recipient’s poorly oxygenated pulmonary arterial circulation through collaterals. Clearly, until neo-vascularization occurs, factors that decrease pulmonary blood flow or increase pulmonary vascular resistance worsen the donor’s bronchial ischemia. These factors include poor graft preservation, lung ischemia–reperfusion injury, severe edema, rejection, infection, inflammation, low cardiac output, hypotension and prolonged positive pressure ventilation^23^.

Donor airway ischemia initially presents with mucosal changes. Progressive ischemia may lead to the necrosis of the bronchial wall and, eventually, dehiscence^24^. Bronchial stenosis is a rather common airway complication, ranging from 1.6% to 32.0%^25,26^. Stenosis may occur on the suture line or it may involve the lobar, segmental and subsegmental bronchi; a recognized non-anastomotic site is the bronchus intermedius. A combination of ischemia and infection is believed to cause the stenotic evolution of the bronchial graft.

The recognition of early mucosal alterations could prove challenging, as these heavily depend on the experience of the single bronchial endoscopist. The recognition and categorization of anastomotic bronchial complications have been the subject of several studies; among these, the Société de Pneumologie de Langue Française (SPLF) developed an endoscopic classification using a standardized and exhaustive grading system in 2013^11^. Such classification includes a macroscopic, diameter and suture (MDS) grading system that suited the purpose of this study. More recently, the International Society for Heart and Lung Transplantation (ISHLT) has elaborated a comprehensive definition for airway complications as well as a grading system^27^. Since according to this classification all ACs observed in our cohort of patients fell into the same category to the authors decided against stratifying the ACs.

Bronchoscopic surveillance protocols are not universally standardized, being mostly institution-related. As previously mentioned, at our center a scheduled protocol is followed^28,29^.

Remarkable enhancements in endoscopic technology have been crucial in the improvement of airways evaluation. Fluorescence bronchoscopy, which was developed as a method for detecting early lung cancers and dysplastic lesions of the tracheobronchial tree, can assess the airway in terms of vascular supply of the bronchial mucosa. AFI is a technology that employs the inherent properties of short wavelength blue light to assess mucosal tissues. When the blue excitation light (390–470 nm) reaches the subepithelial layer of healthy tissue, it will appears green as a consequence of the fluorescence emitted from fluorescent substances such as collagen and light of wavelength 540–560 nm, which is absorbed by circulating hemoglobin. If any subtle mucosal change—such as decreased vascularization or thickening of the mucosa—occurs on the surface layer, fluorescence decreases and the tissue appears as magenta^13^. In light of that, it is legitimate to assume that this technology might help in identifying ischemic bronchi and that, by grading the R/G ratio, it would enable operators to avoid possible anastomotic complications. Not surprisingly, all patients displayed completely red (ischemic) bronchial mucosae at early post-operative checks, although the mucosa appeared pink and completely trophic in white light. By the time the entire study interval passed (12 months), the bronchial mucosa acquired a green tone similar to that of the pre-anastomotic healthy native mucosa. In our cohort, no signs of ischemia, necrosis, or anastomotic dehiscence were detected; conversely, 8 grafts developed post-anastomotic stenosis. It is interesting to note that the “normalization” of the R/G curve diverged significantly from the third postoperative month despite the fact that grafts with post-anastomotic bronchial stenosis had an average complication onset time of 201 days. This evidence opens up the possibility of speculating on the potential predictive capacity of the AFI in terms of development of post-anastomotic bronchial stenoses.

This study has several limitations. The limited number of patients prevents the operator from evaluating every potential anastomotic complication; consequently, only post-anastomotic stenosis can be investigated. Bronchoscopic and AFI procedures were performed by the same operator (RC) and the AFI evaluation is routinely carried out at the first bronchial carina after the bronchial anastomosis, yet little inter-procedure variability, depending on the exact area of the mucosa where the AFI is employed, must be taken into account. Potential bias was also due to the inclusion of lobar LuTx (4 bronchial anastomoses in 3 patients); none of the lobar LuTx patients developed ACs.

The exclusion criteria may be questionable, in particular the ICU stay (longer than 7 days), being that a potential risk factor for ACs. Unfortunately, logistical obstacles prevented the operators from performing AFI in the ICU; therefore, as data on the seventh and/or fourteenth POD was not available, the authors decided against including these patients in the study cohort.

Lastly, this research was further limited by the sample size, in particular that of the ACs group (as absolute number). In order to overcome such limitations, a joint model for statistical analysis was employed.

Nevertheless, this study presents valuable outcomes. To the best of our knowledge, this is the first clinical study that longitudinally analyzes the evolution of the bronchial mucosa after lung transplantation by AFI. Moreover, the examination of the mucosa with autofluorescence does not require any accessory investigation except for the switching on of the light source for a few seconds during the scheduled bronchoscopic controls after LuTx.

In conclusion, AFI is a simple yet efficient imaging technique which improves the control of the bronchial mucosa revascularization after lung transplantation; we observed a likely correlation between post-anastomotic stenosis and delayed R/G ratio decrease. AFI seems to be an effective and manageable diagnostic tool for early diagnosis of ACs after LuTx. Moreover, our preliminary results suggest introducing close endoscopic controls in case of delayed decrease of the R/G ratio at AFI, to provide early diagnoses of ACs and prompt intervention. Further research is needed to confirm and eventually specify the reported preliminary results.
